# The grapevine LysM receptor-like kinase VvLYK5-1 recognizes chitin oligomers through its association with VvLYK1-1

**DOI:** 10.3389/fpls.2023.1130782

**Published:** 2023-02-02

**Authors:** Thibault Roudaire, Tania Marzari, David Landry, Birgit Löffelhardt, Andrea A. Gust, Angelica Jermakow, Ian Dry, Pascale Winckler, Marie-Claire Héloir, Benoit Poinssot

**Affiliations:** ^1^ Agroécologie, CNRS, INRAE, Institut Agro, Univ. Bourgogne, Univ. Bourgogne Franche-Comté, Dijon, France; ^2^ LIPME, Université de Toulouse, INRAE, CNRS, Castanet-Tolosan, France; ^3^ Department of Plant Biochemistry, University of Tübingen, Center for Plant Molecular Biology, Tübingen, Germany; ^4^ Commonwealth Scientific and Industrial Research Organisation (CSIRO), Adelaide, SA, Australia; ^5^ Dimacell Imaging Facility, PAM UMR A 02.102, Institut Agro, Univ. Bourgogne, Univ. Bourgogne Franche-Comté, Dijon, France

**Keywords:** microbe-associated molecular pattern (MAMP)-triggered immunity, pattern recognition receptors (PRR), LysM receptor-like kinase (LYK), Vitis vinifera, Chitooligosaccharides (COS), Chitin (N-acetyl-D-glucosamine), plant immunity

## Abstract

The establishment of defense reactions to protect plants against pathogens requires the recognition of invasion patterns (IPs), mainly detected by plasma membrane-bound pattern recognition receptors (PRRs). Some IPs, also termed elicitors, are used in several biocontrol products that are gradually being developed to reduce the use of chemicals in agriculture. Chitin, the major component of fungal cell walls, as well as its deacetylated derivative, chitosan, are two elicitors known to activate plant defense responses. However, recognition of chitooligosaccharides (COS) in *Vitis vinifera* is still poorly understood, hampering the improvement and generalization of protection tools for this important crop. In contrast, COS perception in the model plant *Arabidopsis thaliana* is well described and mainly relies on a tripartite complex formed by the cell surface lysin motif receptor-like kinases (LysM-RLKs) AtLYK1/CERK1, AtLYK4 and AtLYK5, the latter having the strongest affinity for COS. In grapevine, COS perception has for the moment only been demonstrated to rely on two PRRs VvLYK1-1 and VvLYK1-2. Here, we investigated additional players by overexpressing in Arabidopsis the two putative *AtLYK5* orthologs from grapevine, *VvLYK5-1* and *VvLYK5-2*. Expression of *VvLYK5-1* in the *atlyk4/5* double mutant background restored COS sensitivity, such as chitin-induced MAPK activation, defense gene expression, callose deposition and conferred non-host resistance to grapevine downy mildew (*Erysiphe necator)*. Protein-protein interaction studies conducted *in planta* revealed a chitin oligomer-triggered interaction between VvLYK5-1 and VvLYK1-1. Interestingly, our results also indicate that VvLYK5-1 mediates the perception of chitin but not chitosan oligomers showing a part of its specificity.

## 1 Introduction

Plants are constantly under attack from numerous organisms including fungi, oomycetes, bacteria, viruses, nematodes, insects, and other herbivores. In order to detect invasions and prepare themselves to defend against such pathogens, plants have evolved a sophisticated immune system that relies on the perception of invasion patterns (IPs) by cell receptors (Cook et al., 2015). The first line of plant innate immunity involves cell-surface pattern recognition receptors (PRRs) which harbor different extracellular domains to recognize a wide range of endogenous or exogenous elicitor molecules (Ngou et al., 2022). Following ligand perception, signal transduction inside the cell generally involves a phosphorylation cascade activating mitogen-activated protein kinases (MAPKs), calcium influx, and production of reactive oxygen and nitrogen species (RONS) leading to the activation of transcription factors (Zhou and Zhang, 2020). Depending on the nature and the integration of these signals, appropriate defense mechanisms can then be triggered, such as callose deposition, production of phytoalexins and pathogenesis-related (PR) proteins, stomatal and plasmodesmal closure, and eventually a programmed cell death (Yu et al., 2017; Roudaire et al., 2021).

Chitin, a component of fungal cell walls, insects’ and crustaceans’ cuticles, as well as nematode eggs, and chitosan, its deacetylated form, are two examples of IPs also referred to as chitooligosaccharides (COS). These microbe-associated molecular patterns (MAMPs) are perceived by the lysin motif (LysM) receptor-like kinase (RLK) class of PRRs. The plasma membrane (PM)-localized LysM-RLKs are categorized into two main sub-groups depending on whether they harbor a kinase domain, either functional or not or a glycosylphosphatidylinositol (gpi)-anchor (LYMs) (Buendia et al., 2018). They can perceive a wide range of polysaccharides also including peptidoglycans (Willmann et al., 2011; Liu et al., 2012a), exopolysaccharides (Kawaharada et al., 2015), nodulation and mycorrhization factors (Amor et al., 2003; Girardin et al., 2019; He et al., 2019; Zhang et al., 2019), and mixed-linkage glucans (Barghahn et al., 2021). Ligand perception often involves the association of receptors into supra-molecular complexes. Moreover, PRRs, especially those lacking a functional kinase domain, must interact with other co-receptors to transduce the signal across the plasma membrane inside the cell. For chitin oligomers, the perception mechanism has been well described in the model plant *Arabidopsis thaliana*. In this plant species, AtLYK5, a LysM-RLK having a strong affinity for chitin is proposed to form homodimers or heterodimers with AtLYK4 upon ligand-binding (Wan et al., 2012; Cao et al., 2014; Xue et al., 2019). As neither of these two receptors are capable of signal transduction, AtLYK5 must interact with AtCERK1/LYK1, a ubiquitous LysM-RLK which has a low affinity for chitin oligomers but possesses a functional kinase domain (Miya et al., 2007; Cao et al., 2014). A similar model of molecular complexes has been proposed for the chitin perception in rice involving the LysM-RLK OsCERK1 and the LYM proteins OsCEBIP, OsLYP4 and OsLYP6 (Shimizu et al., 2010; Liu et al., 2012a; Liu et al., 2013; Yang et al., 2022).

To date, only the two orthologs of AtCERK1/LYK1, VvLYK1-1 and VvLYK1-2, have been shown to mediate the perception of COS in grapevine (*Vitis vinifera*) and to restore COS-mediated MAPKs activation and defense gene expression in the *Arabidopsis atlyk1* mutant (Brulé et al., 2019; Héloir et al., 2019). Here, we aimed at understanding the function of *AtLYK5* orthologs in grapevine as well as their involvement in the resistance to fungal pathogens. Using functional complementation of the Arabidopsis *atlyk4/5* double mutant, which is completely deficient in COS-triggered immune responses, we demonstrated that *VvLYK5-1* is involved in chitin-induced immunity in *Vitis vinifera*, contrary to *VvLYK5-2* whose function remains to be clarified. Moreover, chitin-dependent interaction between VvLYK5-1 and VvLYK1-1 was demonstrated by Förster resonance energy transfer coupled with fluorescence lifetime imaging microscopy (FRET-FLIM). Finally, VvLYK5-1 and VvLYK5-2 were both found to participate in basal resistance against grapevine powdery mildew *Erysiphe necator* when overexpressed in *A. thaliana*.

## 2 Materials and methods

### 2.1 Phylogenetic analysis of the VvLYK family

The phylogenetic tree was inferred by using the Maximum Likelihood method and JTT matrix-based model (Jones et al., 1992) with a bootstrap of 1000 replications. The tree with the highest log likelihood (-28734.46) is shown. The percentage of trees in which the associated taxa clustered together is shown next to the branches. Initial tree(s) for the heuristic search were obtained automatically by applying Neighbor-Join and BioNJ algorithms to a matrix of pairwise distances estimated using a JTT model, and then selecting the topology with superior log likelihood value. The tree is drawn to scale, with branch lengths measured in the number of substitutions per site. This analysis involved 22 amino acid sequences. There were a total of 1230 positions in the final dataset. Phylogenetic analysis was conducted in *MEGA X* (Kumar et al., 2018).

### 2.2 Expression analysis of *VvLYK* genes in pathogen-infected grapevine tissues

RNAseq expression data on *VvLYK* genes were extracted from public data hosted on the NCBI website (https://www.ncbi.nlm.nih.gov/). *VvLYK* expression profiles of *Vitis vinifera* cv. Carignan leaf tissues following an infection with *Erysiphe necator* were extracted from the list of differentially expressed genes from Amrine et al. (2015) (BioProject Accession: PRJNA279229). *VvLYK* expression profiles of *V. vinifera* cv. Victoria leaf tissues following an infection with *Coniella diplodiella* were extracted from the study of Su et al. (2019) (BioProject Accession: PRJNA476839) using the GRape Expression ATlas (*GREAT)* application (https://great.colmar.inrae.fr/).

### 2.3 Plant materials and elicitors

*Arabidopsis thaliana* wild-type (WT) Columbia (Col-0), *atlyk4* mutant (WiscDsLox297300_01C), *atlyk5* mutant (SALK_131911C, allele *atlyk5-2*), *atlyk4/5* double mutant (WiscDsLox297300_01C x SALK_131911C; Cao et al., 2014) or transgenic lines *atlyk4/5-p35S::VvLYK5-1/-2* were grown under a 10/14-h day/night cycle at 20/18°C. Transgenic lines were obtained by floral-dip transformation (Clough and Bent, 1998) of the *atlyk4/5* double mutant line with coding sequences of *VvLYK5-1* or *VvLYK5-2*, amplified from complementary DNA (cDNA) of *Vitis vinifera* cv. Marselan leaves and cloned in the pFAST_R02 overexpression vector (Shimada et al., 2010). Transgenic seeds were selected by their red fluorescence using the n°63-HE filter on a Zeiss Axio Zoom V16 microscope. Homozygous seeds of the T3 generation were genotyped before use in the experiments thereafter (**Figure S1A**).

*V. vinifera* cv. Marselan cells were cultivated, under continuous light, in liquid Nitsch-Nitsch medium (Nitsch and Nitsch, 1969) supplemented with 1 g/L casein hydrolysate, 400 µg/L 1-naphthaleneacetic acid and 40 µg/L 6-benzylaminopurine. They were maintained in suspension by continuous shaking (120 rpm at 24°C) and subcultured every 7 days using a 1:6 dilution in 120 mL of fresh liquid medium. For the experiments, grapevine cells were used 24h after diluting twice the 7-day-old culture in fresh medium.

Purified hexamer (DP6) of chitin (*GLU436*) and chitosan (*GLU426*) were provided by Elicityl (Crolles, France). These chitooligosaccharides were dissolved in sterile ultrapure water and used at a final concentration of 0.1 g/L in all experiments.

### 2.4 MAPK activation

Leaves of 4 weeks old Arabidopsis plants were cut, pre-infiltrated with ultrapure water then equilibrated, abaxial face on ultrapure water, for 4h in a 6-well plate. They were then treated by substitution of water with elicitors or water (mock treatment) and harvested 10 min after. Proteins were extracted using a buffer containing 50 mM Hepes (pH 7.5), 5 mM EGTA (pH 8.1), 5 mM EDTA, 1 mM Na_3_VO_4_, 50 mM β-glycerophosphate, 10 mM NaF, 1 mM phenylmethanesulfonyl fluoride, 5 mM dithiothreitol, and 1X cOmplete™ EDTA-free Protease Inhibitor Cocktail (Roche). MAPKs activation was detected after immunoblotting of the extracted proteins (20 µg) using an anti-p42/44-phospho-ERK antibody (Cell Signaling). The revealing step was performed on an Amersham™ ImageQuant™ 800 (Cytiva) using ECL™ Prime as a western blotting detection reagent. Transfer quality and homogeneous loading were checked by Ponceau red staining. Three independent experiments were performed.

### 2.5 Real-time quantitative reverse-transcription polymerase chain reaction (qPCR)

*Arabidopsis thaliana* leaves were treated in the same way as for assessing MAPK activation and harvested 1h post-treatment (hpt). After grinding, total RNAs were extracted using the SV Total RNA Isolation System with DNAse treatment (Promega). First-strand cDNA was synthesized from 1 μg of total RNA using the High Capacity cDNA Reverse Transcription kit (Applied Biosystems). Real-time qPCR was performed in a ViiA™ 7 Real-Time PCR system (Applied Biosystems) with 10 ng cDNA and GoTaq^®^ qPCR Master Mix (Promega). Relative gene expression was assessed according to the Common Base Method (Ganger et al., 2017) taking into consideration the efficiency (E) of each reaction calculated by the LinRegPCR quantitative PCR data analysis program (Ruijter et al., 2009). For each gene of interest (GOI), the mean of resulting technical duplicate data (efficiency-weighted ${\text{C}}_{q}^{\left(\text{w}\right)}$ values = *C*_*q*_×log(*E*) ) was normalized by the mean ${\text{C}}_{q}^{\left(\text{w}\right)}$ data of *AtRHIP1* (*AT4G26410*) and *AtPTB1* (*AT3G01150*) housekeeping genes (HKG) ( $\Delta C_{q}^{\left(w\right)}=\;C_{q;\;GOI}^{\left(w\right)}-\frac{C_{q;\;HKG_{1}}^{\left(w\right)}+\;C_{q;\;HKG_{2}}^{\left(w\right)}}{2}$ ) before being normalized to the water control treatment (Fold-change = $10^{-\Delta \Delta C_{q}^{\left(w\right)}}$ , with $\Delta \Delta C_{q}^{\left(w\right)}=\;\Delta C_{q;\;COS}^{\left(w\right)}-\Delta C_{q;\;Mock}^{\left(w\right)}$ ). All primers used are listed in **Table S1**. Four independent experiments were conducted.

For grapevine cells, suspensions were divided into 100 mL flasks then equilibrated for 1.5h on a rotary shaker with the same conditions as for their culture. They were next treated with chitin (final concentration of 0.1 g/L in a final volume of 20 mL) or water (as control) and harvested at 1h, 3h, 6h, and 24h post-treatment (hpt). The qPCR protocol and calculation method were the same as described above except that *VvVATP16* (*Vitvi03g04022*), *VvRPL18B* (*Vitvi05g00033*) and *VvVPS54* (*Vitvi10g01135*) were used as housekeeping genes. Five independent experiments were conducted.

### 2.6 Callose deposition detection

Four-week-old Arabidopsis plants were sprayed on both sides with a solution of chitin or water as control. Four days post-treatment, one leaf from each of the two plants used for each modality was harvested and bleached in pure ethanol. They were then cleared overnight in 1 g/L chloral hydrate and washed three times with 0.1 M phosphate buffer (pH 8.0) before being stained in 0.1 M phosphate buffer (pH 8.0) containing 0.01% (w/v) aniline blue for a day. After staining, leaves were mounted on slides in 80% (v/v) glycerol. Callose deposition was observed on the adaxial side by epifluorescence microscopy under UV (λ_exc_ = 340-380 nm, λ_em_ = 425 nm, long pass filter, magnification x100, Leica DMRB). Ten representative images of the callose deposits were acquired for each condition using the Nis Elements BR software (Nikon) with the DS-5Mc-U1 digital photomicrographic camera (Nikon) equipped on the microscope. Image analysis was then performed on the Fiji application (Schindelin et al., 2012) as described in Mason et al. (2020). Three independent experiments were conducted.

### 2.7 Pathogen assays

*Erysiphe necator* was grown on detached leaves of *V. vinifera* cv. Marselan previously disinfected in a solution of 2% (v/v) sodium hypochlorite, maintained on sterile agar plates and subcultured every two weeks. Four-week-old *A. thaliana* plants were used to assess powdery mildew penetration efficiency. Two leaves per plant were infected with *E. necator* spores using a fine paintbrush. Detached leaves from two plants per line were sampled 48h post-inoculation (hpi) and stained with trypan blue according to Koch and Slusarenko (1990). Fungal structures were visualized using a Leica (Wetzlar, Germany) DME light microscope (magnification x400). A minimum of 100 germinated spores were scored for each treatment. Successful penetration of epidermal cells (% of penetrated cells) was indicated by the presence of a haustorium within the cell or the development of a secondary hyphae from the appressorium as described in **Figure S2**. Three independent experiments were performed with the same results.

### 2.8 Subcellular localization and protein immunoblotting

Leaves of *Nicotiana benthamiana* were transformed by agroinfiltration (Norkunas et al., 2018) with the *Agrobacterium tumefaciens* strain *GV3101* containing an overexpression cassette for the expression of *VvLYK5-1_YFP* or *VvLYK5-2_YFP* (see below), *MtNFP_mCherry* (coding for a protein previously described to be localized at the PM; Lefebvre et al., 2012), and the RNA silencing suppressor *p19* (Voinnet et al., 2003), with OD600 of each strain adjusted to 0.5, 0.5 and 0.2 respectively. After 72 hours, the subcellular localization of fusion proteins was studied and 3 leaf discs (7 mm of diameter) were harvested for immunoblot analysis. Subcellular localization was analyzed with a confocal laser scanning microscope (SP8, Leica) using a x25 water immersion objective lens. Leaf discs were ground using a Restch MM400 mixer mill. Total proteins were extracted in 2X Laemmli buffer and boiled at 95°C for 5 minutes and separated on SDS-PAGE, and then transferred to a nitrocellulose membrane. Immunodetection of the fusion proteins (**Figure S3**) was performed using the rabbit anti-GFP (Sigma-Aldrich) and anti-mCherry polyclonal antibodies (Lefebvre et al., 2012), and polyclonal goat anti-rabbit antibodies fused to HRP (Millipore).

### 2.9 FRET-FLIM analysis

Leaves of *Nicotiana tabacum* cv. Xanthi were transformed by agroinfiltration (Norkunas et al., 2018) with the *Agrobacterium tumefaciens* strain *GV3101* containing an overexpression cassette for VvLYK1-1, VvLYK1-2, VvLYK5-1, or VvLYK5-2 fused with either a C-terminal Cyan Fluorescent Protein (C_ter_-CFP in the pH7CWG2 plasmid, Karimi et al., 2005) or a C-terminal Yellow Fluorescent Protein (C_ter_-YFP in the pH7YWG2 plasmid, Karimi et al., 2005). Five days after (co-)infiltration, the abaxial side of *N. tabacum* leaves transiently expressing the different constructs was infiltrated with chitin or water (mock treatment). Observations were performed 30 min post-treatments (according to Cheval et al., 2020) using a Nikon A1-MP multiphoton microscope with an Apo IR x60 objective (NA: 1.27, water immersion, Nikon). Fluorescence lifetime imaging (FLIM) images were collected using a time-correlated single-photon counting (TCSPC) module (Picoquant). CFP excitation (820 nm with two-photon excitation) was provided by an IR laser (Chameleon, Coherent) delivering femtosecond pulses at a repetition rate of 80 MHz. Its resulting fluorescence emission was collected with a single photon avalanche diode (SPAD), using a band-pass emission filter FF01-494/20 (Semrock). TCSPC lifetime recording was performed over 200 temporal channels (final resolution: 0.64 ps). The FLIM analysis was performed on regions of interest (ROIs) drawn on the plasma membrane using the SymPhoTime (PicoQuant) software. Fluorescence lifetime values were calculated by fitting the tail of the CFP fluorescence decay with a bi-exponential model. Among the two generated lifetime constants (τ_1_ and τ_2_), only τ_1_ was reported, as it was the most sensitive to the Förster resonance energy transfer (FRET) (Bègue et al., 2019; Rosnoblet et al., 2021). The efficiency of the energy transfer was given by the following equation: $E\left(\%\right)=\left(1-\frac{F_{DA}}{F_{D}}\right)\times 100$ , with FDA and FD representing the relative fluorescence lifetime of the donor in the presence or the absence of the acceptor, respectively. Three independent experiments were conducted.

## 3 Results

### 3.1 Identification and characterization of grapevine orthologs to AtLYK5

**Figure 1:**
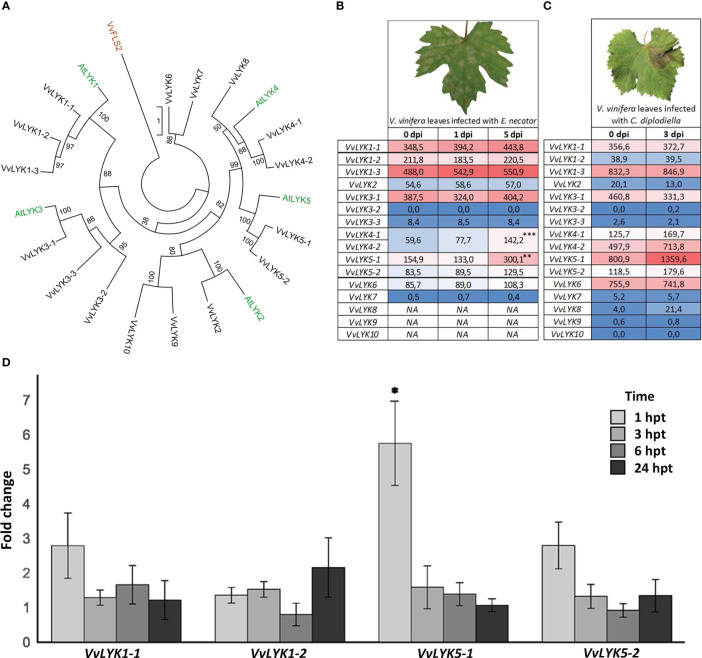
Phylogenetic analysis and expression profiles of grapevine LysM-RLK genes (*VvLYKs)*. **(A)** The maximum-likelihood phylogenetic tree constructed with MEGA X (Kumar et al., 2018) shows the relationship between Arabidopsis (green) and grapevine (black) LYK families. Protein sequences used for the phylogenetic analysis were: VvLYK1-1 (Vitvi12g00415), VvLYK1-2 (Vitvi10g00050), VvLYK1-3 (Vitvi10g01613), VvLYK2 (Vitvi14g02422), VvLYK3-1 (Vitvi09g00283), VvLYK3-2 (Vitvi04g00472), VvLYK3-3 (Vitvi01g02041), VvLYK4-1 (Vitvi04g01218), VvLYK4-2 (Vitvi04g01216), VvLYK5-1 (Vitvi18g00013), VvLYK5-2 (Vitvi18g00014), VvLYK6 (Vitvi05g00623), VvLYK7(Vitvi04g01214), VvLYK8 (Vitvi18g00904), VvLYK9 (Vitvi17g00941), VvLYK10 (Vitvi06g00251), AtLYK1(AT3G21630), AtLYK2 (AT3G01840), AtLYK3 (AT1G51940), AtLYK4 (AT2G23770), AtLYK5 (AT2G33580).VvFLS2 (Vitvi10g00742) was used to root the tree. **(B)** *VvLYKs* expression profiles in *Vitis vinifera* leaf tissues following infection with *Erysiphe necator*. Data are normalized counts derived from the publicly available dataset of Amrine et al. (2015). Asterisks indicate statistically up-regulated genes with a log2(Fold-change) ≥ 0.5 (p-values adjusted to mean infected/uninfected normalized counts; ***, P< 0.001; **, P< 0.01). *VvLYK4-1/-2* expression data could not be separated as they were previously annotated as the same gene. NA indicates missing data (not detected or non-annotated). **(C)** *VvLYKs* expression profiles from *V. vinifera* leaf tissues following infection with *Coniella diplodiella*. Data are RPKM counts derived from the publicly available dataset of Su et al. (2019) and processed using the GREAT application. **(D)** Fold change in gene expression of *VvLYK1-1*, *VvLYK1-2*, *VvLYK5-1* and *VvLYK5-2* measured by qPCR 1h, 3h, 6h and 24h after chitin treatment (0.1 g/L) of *V. vinifera* cv. Marselan cell suspensions. Data represent the normalized mean fold-change ± SE from five independent experiments. Asterisks indicate a statistically significant difference to the water control, set as 1 (Kruskal-Wallis with Dunn *post-hoc* test; *, P< 0.05 after BH p-value adjustment).

In contrast to *A. thaliana* that possesses 5 LysM-RLKs (Wan et al., 2008), the most recent annotation of the grapevine genome predicts 16 *VvLYK* genes encoding 16 LysM-RLKs. To narrow down the number of candidates for COS perception, we performed a phylogenetic analysis of this multigene family in these two species. An actualized maximum-likelihood phylogenetic tree was constructed in order to highlight protein sequence similarities between grapevine and Arabidopsis LYKs (**Figure 1A**). Two sequences, named VvLYK5-1 and VvLYK5-2 according to the previous naming convention proposed by Brulé et al. (2019), were found unambiguously located in the same clade as the major Arabidopsis chitin receptor AtLYK5 (Cao et al., 2014) since they were clustered together in 100% of the trees constructed during the analysis (**Figure 1A**).

Expression profiles following inoculation with fungal pathogens of each predicted *VvLYK* gene were then analyzed using publicly available RNAseq data (Amrine et al., 2015; Su et al., 2019) to identify genes that are transcriptionally regulated during the plant defense reaction against these chitin-containing organisms. **Figures 1B, C** indicate that *VvLYK5-1* was up-regulated in response to infection by both *E. necator* and *Coniella diplodiella* (grapevine white rot). Interestingly, neither *VvLYK5-2* nor the previously identified genes *VvLYK1-1* and *VvLYK1-2* encoding COS receptors (Brulé et al., 2019) appeared to be differentially expressed in grapevine leaves in response to these two pathogens. To confirm publicly available transcriptomic data, grapevine cells were treated with purified chitin hexamer (DP6, thereafter referred as chitin treatment). Our results confirmed that only *VvLYK5-1* transcripts were significantly upregulated 1h post-treatment (**Figure 1D**).

**Figure 2:**
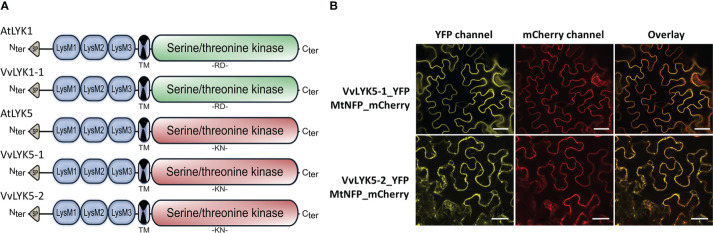
Protein domains and subcellular localization of VvLYK5-1 and VvLYK5-2. **(A)** Schematic representation of protein structure of AtLYK1, VvLYK1-1, AtLYK5, VvLYK5-1, and VvLYK5-2 based on multiple alignments using Clustal W (**Figure S4**). Similar to their Arabidopsis orthologs, VvLYK1-1 possesses a functional kinase domain (RD type in green) whereas VvLYK5-1 and VvLYK5-2 do not (non-RD type in red). SP, signal peptide; LysM: Lysin motif; TM, transmembrane. **(B)** Subcellular localization of VvLYK5-1 and VvLYK5-2 in *Nicotiana benthamiana* leaves. VvLYK5-1_YFP fully co-localized with the plasma membrane marker (PM) MtNFP_mCherry. VvLYK5-2_YFP co-localized with the PM marker but also appeared partly in the endoplasmic reticulum. Scale bar indicates 50 μm.

Further sequence analysis of VvLYK5-1 and VvLYK5-2 was performed after cloning and sequencing their respective cDNAs from leaves of *V. vinifera* cv. Marselan. *In silico* analysis revealed the presence of a signal peptide, three extracellular LysM domains, a transmembrane domain, and a cytoplasmic serine/threonine kinase domain (**Figure 2A**, **Figure S4**). As expected, VvLYK5-1_YFP was detected at the PM (**Figure 2B**) as it co-localized with the PM marker MtNFP_mCherry (Lefebvre et al., 2012). VvLYK5-2_YFP mainly accumulated at PM and also appeared partly in the endoplasmic reticulum (**Figure 2B**). Moreover, the analysis of the predicted amino acid sequences of VvLYK5-1 and VvLYK5-2 (**Figure S4**) indicated that most of the amino acids known to be important for the kinase activity (Hanks et al., 1988; Johnson et al., 1996; Petutschnig et al., 2010; Klaus-Heisen et al., 2011; Suzuki et al., 2016) were absent, as in AtLYK5 (Cao et al., 2014). Thus, in the Gly-rich loop, three out of the four glycines present in AtLYK1/VvLYK1-1 and MtLYK3 were absent in VvLYK5-1/-2, as in AtLYK5. Similarly, in the VAIKK motif, the ATP binding site K^350^ of AtLYK1 (X^451^ of the consensus sequence) which is mandatory for the kinase activity (Petutschnig et al., 2010), was mutated in AtLYK5 and VvLYK5-1/-2. In the catalytic loop, the amino acids RD found in AtLYK1, VvLYK1-1 and MtLYK3 were replaced by the KN residues in AtLYK5, VvLYK5-1 and VvLYK5-2. This difference is crucial knowing that a single mutation of D^441^ in MtLYK3 (X^545^ on the consensus sequence) leads to a loss of its *in vitro* kinase activity (Klaus-Heisen et al., 2011). In the activation segment, the aspartic acid of the DFG motif, that is well conserved across kinases (Hanks et al., 1988), is also mutated in AtLYK5, VvLYK5-1 and VvLYK5-2. Finally, the phosphorylation site T^573^ of AtLYK1 (X^686^ of the consensus sequence), required for the activity of the AtLYK1 kinase domain (Suzuki et al., 2016) and also conserved in MtLYK3 (Klaus-Heisen et al., 2011), was absent in the two VvLYK5-1/-2 protein sequences. When considered together, all of these differences suggest that VvLYK5-1 and VvLYK5-2 do not possess a functional kinase domain.

### 3.2 *VvLYK5-1* but not *VvLYK5-2* restores chitin-induced early defense events in the *atlyk4/5* double mutant

In order to compare the two related elicitors based on their degree of acetylation (DA), we chose to use COS with the same degree of polymerization (DP). Since COS with a low DP (*i.e.* from 6 to 8 glucosamine (GlcN) or N-acetyl-glucosamine (GlcNAc) residues) are known to induce the most effective defense reactions in different plant species, including grapevine and Arabidopsis (Aziz et al., 2006; Miya et al., 2007; Petutschnig et al., 2010; Brulé et al., 2019), we also used COS with this DP in this study.

**Figure 3:**
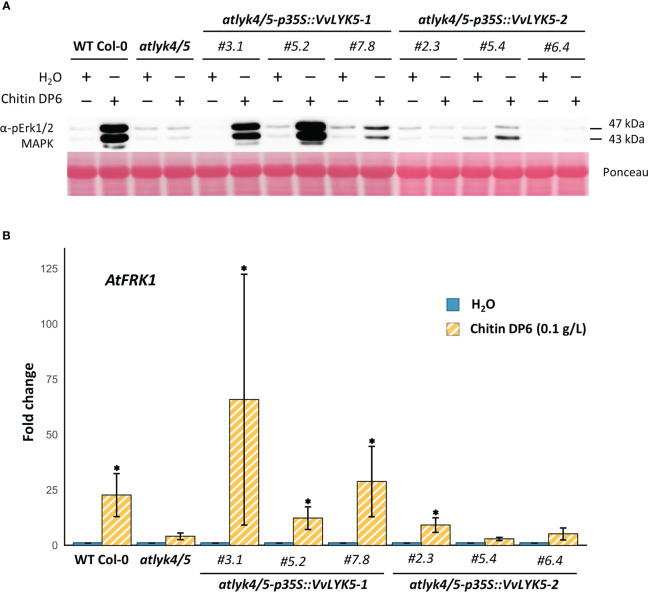
VvLYK5-1 restores early chitin-induced immune responses in the *Arabidopsis thaliana lyk4/5* double mutant background. **(A)** The activation of mitogen-activated protein kinases (MAPKs) was detected 10 min after chitin treatment (0.1 g/L) by immunoblotting with an antibody raised against the human phosphorylated extracellular regulated protein kinase 1/2 (Erk1/2). Equal protein loading was confirmed by Ponceau S red staining. Similar results were obtained in three independent experiments. **(B)** Fold change in gene expression of flagellin-induced receptor kinase 1 (*AtFRK1*; *At2g19190*) measured by qPCR 1h after chitin (0.1 g/L) or water treatment. Data represent the mean fold-change ± SE from four independent experiments. Means of technical duplicates (efficiency-weighted Cq(w) values) were normalized using mean Cq(w) data of two housekeeping genes (*AtRHIP*1 and *AtPTB1*) before being normalized to the control treatment. Asterisks indicate a statistically significant difference with the water control (Kruskal-Wallis with Dunn *post-hoc* test; *, P< 0.05 after BH p-value adjustment). WT Col-0, wild-type Columbia-0 ecotype.

To find out whether VvLYK5-1 or VvLYK5-2 were able to trigger immune responses in response to COS, we overexpressed their encoding genes in the *atlyk4/5* double mutant, known to be insensitive to the chitin oligomers (Cao et al., 2014). Three independent lines of each construct overexpressing *VvLYK5-1* or *VvLYK5-2* (**Figures S1B, C**) were first evaluated on their ability to induce MAPKs activation in response to a chitin oligomer treatment. As expected, chitin induced the phosphorylation of two MAPKs, MPK3 and MPK6 (at 43 and 47 kDa, respectively) in WT Col-0 plants (Cao et al., 2014) but not in the *atlyk4/5* double mutant (**Figure 3A**). This chitin-induced MAPKs activation was restored in the three transgenic *atlyk4/5-p35S::VvLYK5-1* lines. Weaker response in line #7.8 was correlated with lower transgene expression (**Figure S1B**). In contrast, MAPKs activation was not restored in transgenic *atlyk4/5-p35S::VvLYK5-2* lines (**Figure 3A**). To note, a weak chitin-induced activation of MPK3 in *atlyk4/5-p35S::VvLYK5-2* lines could be detected in only one out of three experiments and was therefore not considered. Next, the expression of the defense gene encoding *flagellin-induced receptor kinase 1* (*FRK1*), which has been previously shown to be a chitin-responsive gene (Brulé et al., 2019), was monitored. **Figure 3B** shows that *AtFRK1* gene expression was transcriptionally upregulated upon chitin treatment in WT Col-0 plants but not in the *atlyk4/5* line. Overexpression of *VvLYK5-1* in the *atlyk4/5* mutant line restored *AtFRK1* expression in each of the three evaluated transgenic lines (**Figure 3B**). In contrast, overexpression of its paralogous gene, *VvLYK5-2*, resulted in a weak chitin-triggered *AtFRK1* expression in only one of the three evaluated lines (**Figure 3B**).

The *atlyk4/5* double mutant was also unable to elicit defense responses after chitosan hexamer treatment (**Figure S5**). Interestingly, in transgenic lines overexpressing *VvLYK5-1* or *VvLYK5-2*, chitosan treatment did not appear to clearly restore the MAPKs activation compared to WT (**Figure S6A**), and the level of *AtFRK1* transcripts was not statistically different compared to water treatment (**Figure S6B**). In sum, these results suggest that *VvLYK5-1* can complement chitin-triggered immune responses in the *atlyk4/5* double mutant line, but probably not those triggered by chitosan. Moreover, *VvLYK5-2* does not seem to play a major role in grapevine for the perception of chitin or chitosan oligomers.

### 3.3 *VvLYK5-1* restores chitin-mediated callose deposition in the *atlyk4*/5 double mutant

**Figure 4:**
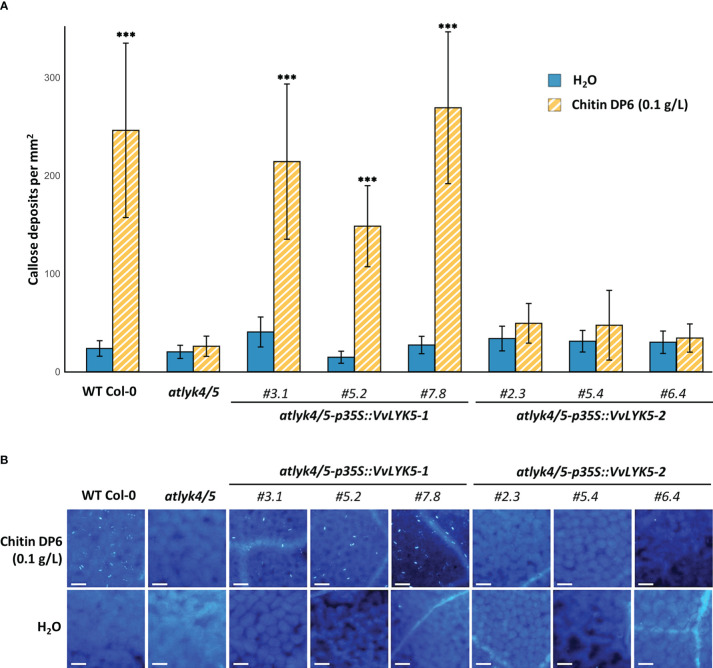
VvLYK5-1 restores chitin-mediated callose deposition in the *atlyk4/5* double mutant. Callose deposition was analyzed by epifluorescence microscopy after aniline blue staining 4 days post-treatment with chitin (0.1 g/L) or water control. **(A)** Callose deposition was presented as the mean callose deposits observed per area (1 mm²) ± SE from 3 independent experiments, each with 10 representative pictures per condition. Asterisks indicate a significant difference with the *atlyk4/5* double mutant line (Two-way ANOVA with Tukey *post-hoc* test; ***, P< 0.001). **(B)** Representative images of callose deposits induced in each condition. Scale bar indicates 50 µm.

As chitin is known to induce callose deposition in *A. thaliana* leaves (Giovannoni et al., 2021), we investigated whether the expression of *VvLYK5-1* could also complement this late defense response event. **Figure 4** indicates that the *atlyk4/5* double mutant did not accumulate callose deposits in response to chitin treatment. Indeed, the number of callose deposits, close to 200 per mm² in the WT in response to chitin treatment, decreased to a level similar to a mock treatment in the *atlyk4/5* double mutant. This is in agreement with previous reports showing that *atlyk4* and *alyk5* mutants do not trigger any chitin-mediated callose deposition in plasmodesmata (Cheval et al., 2020). By evaluating our transgenic lines, we could observe that *VvLYK5-1* overexpression was sufficient to restore the chitin-induced callose deposition in the *atlyk4/5* double mutant (**Figure 4**). Levels of callose deposition similar to the WT were detected in all the three independent *atlyk4/5-p35S::VvLYK5-1* lines with a number of deposits per mm² ranging from 100 to 300 depending on the transgenic line and not in the three independent *atlyk4/5-p35S::VvLYK5-2* lines (**Figure 4**). Together, these data indicate that only the expression of *VvLYK5-1* can restore this chitin-induced late defense response in the *atlyk4/*5 double mutant.

### 3.4 *VvLYK5-1* and *VvLYK5-2* overexpression restore penetration resistance against the non-adapted powdery mildew in the *atlyk4*/5 double mutant

**Figure 5:**
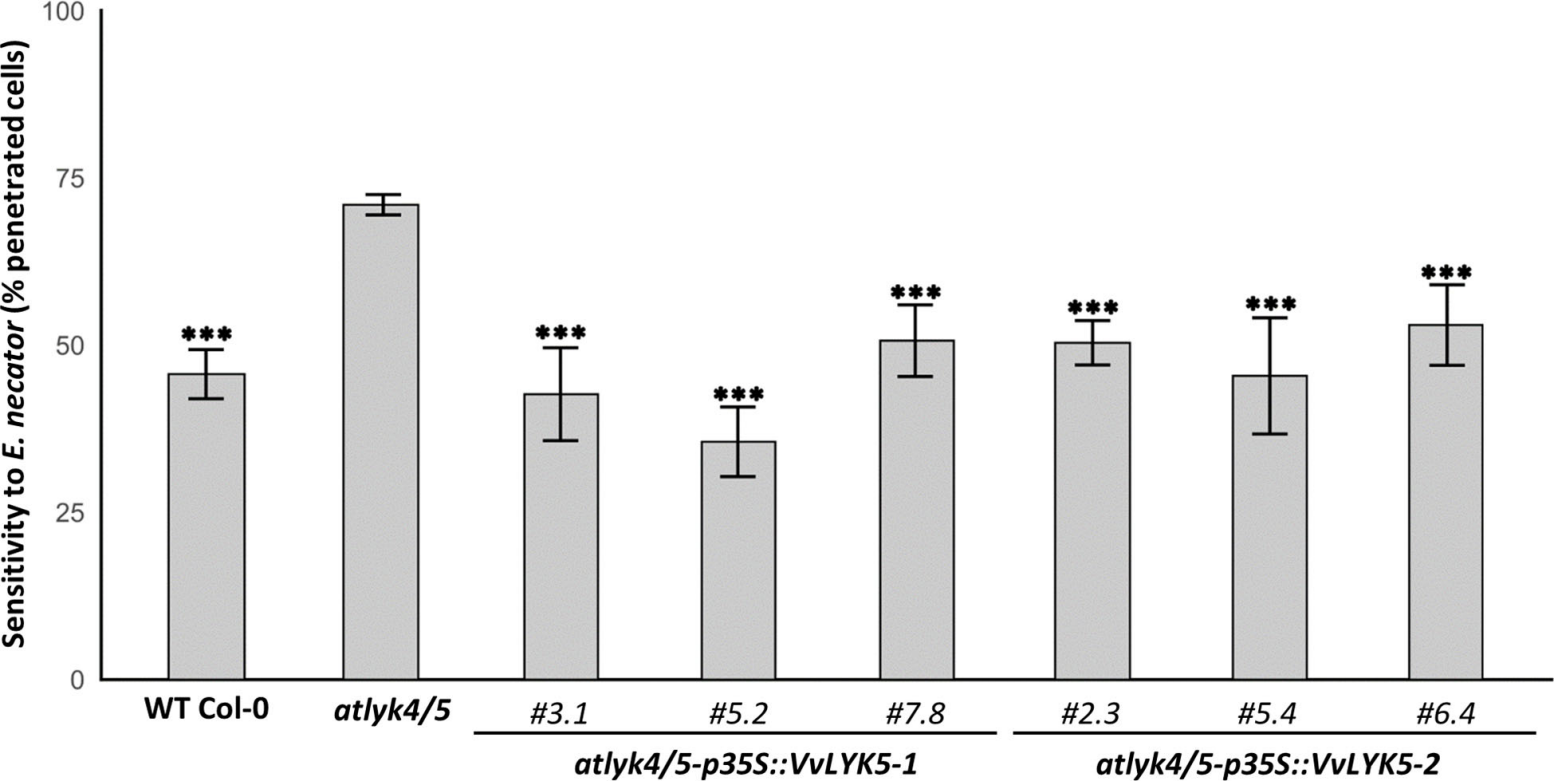
VvLYK5-1 and VvLYK5-2 restore penetration resistance against the non-adapted powdery mildew *E. necator* in the *atlyk4/5* double mutant. Penetration efficiency (*i.e.* haustorium formation) of the non-adapted powdery mildew pathogen *E. necator* on Arabidopsis WT (Col-0), *atlyk4/5* double mutant, and three independent transgenic lines of *atlyk4/5* transformed with overexpression cassettes of *VvLYK5-1* or *VvLYK5-2*. One hundred germinated conidia were scored on two plants per line for each experiment. Each data point represents the mean of three independent experiments ± SE. WT Col-0 and transgenic lines were compared to the double mutant *atlyk4/5* (Pairwise comparison of proportions; ***, P< 0.001 after holm p-value adjustment). The scoring method is detailed in **Figure S2**.

We have previously demonstrated that VvLYK1-1 plays an important role in host resistance to penetration by a non-adapted powdery mildew species (Brulé et al., 2019). We therefore next investigated whether also VvLYK5-1 or VvLYK5-2 are required for host resistance to powdery mildew penetration. First, we evaluated if the *atlyk4/5* double mutant line was more sensitive to powdery mildew than the WT, as it was previously reported for *atcerk1/lyk1* (Brulé et al., 2019). Our results indicated that the *atlyk4/5* double mutant was significantly more sensitive to the non-adapted fungus *E. necator* than the WT Col-0, with a percentage of epidermal penetrated cells increasing from 45% in the WT to 71% in the double mutant (**Figure 5**). However, penetration resistance was restored back to WT levels in *atlyk4/5* mutant lines overexpressing *VvLYK5-1.* This observation suggests that VvLYK5-1 might be also important for grapevine defense against powdery mildew. Remarkably, this restored resistance to the Arabidopsis non-adapted pathogen was also found in the three independent transgenic lines *atlyk4/5-p35S::VvLYK5-2* (**Figure 5**).

### 3.5 VvLYK5-1 interacts with VvLYK1-1 after chitin treatment

Browsing through the *STRING* database (Szklarczyk et al., 2019) to identify putative VvLYK5-1 and VvLYK5-2 interacting partners, we found that both were predicted to interact with VvLYK1-1, VvLYK1-2, and VvLYK1-3 according to reported interactions from orthologous proteins in other species (**Figure S7**). Based on the findings that (a) AtLYK1 is known to directly interact with AtLYK5 for chitin binding (Cao et al., 2014), (b) VvLYK5-1 is probably lacking a functional kinase domain (**Figure S3**), and (c) VvLYK5-2 (this study) and VvLYK1-3 (Brulé et al., 2019) are probably not involved in COS-triggered immunity, we hypothesized that putative interactions might take place between VvLYK5-1 and VvLYK1-1 or VvLYK1-2 for COS perception.

According to basal expression data obtained from *Grape eFP Browser* (Fasoli et al., 2012), it is unlikely that VvLYK5-1 and VvLYK1-2 can interact as *VvLYK5-1* is globally well expressed in all tissues/organs whereas *VvLYK1-2* is not (*e.g*. in adult leaves) or weakly expressed except in wooden stems and flowers (**Figure S8**). In contrast, *VvLYK1-1* and *VvLYK5-1* show high co-expression levels in the whole plant (**Figure S8**) which is consistent with a putative function of VvLYK1-1 as the main co-receptor interacting with VvLYK5-1.

**Figure 6:**
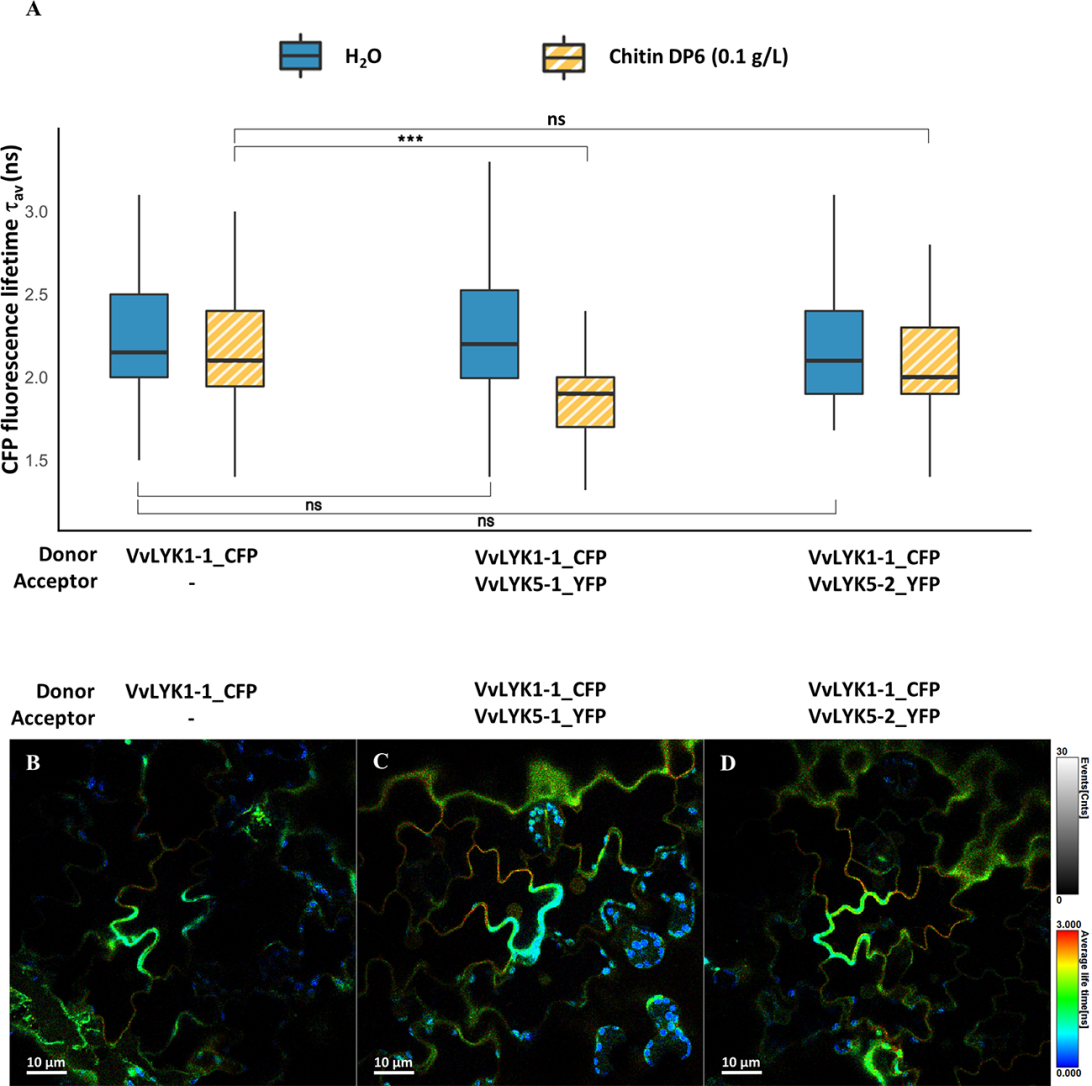
VvLYK5-1 associates with VvLYK1-1 after chitin treatment. **(A)** The fluorescence lifetime of CFP fused to VvLYK1-1 was measured in tobacco leaves (co-) expressing indicated constructs as donor or acceptor, 30 min after infiltration of chitin (0.1 g/L) or water as a control. Box plots represent CFP fluorescence-weighted average lifetime (τav); the box signifies upper and lower quartiles, the line within the box marks the median, and the whiskers represent the maximum and minimum within the 1.5 x interquartile range. Asterisks indicate a statistical significance with the donor alone, with respect to the treatments. The number of ROIs (N) analyzed was ≥ 50 (Two-way ANOVA with Tukey *post-hoc* test; ***, P< 0.001). **(B–D)** Representative FLIM images of **(B)** VvLYK1-1_CFP, **(C)** VvLYK1-1_CFP + VvLYK5-1_YFP, and **(D)** VvLYK1-1_CFP + VvLYK5-2_YFP, all taken 30 min after chitin treatment. The vertical scale bar on the right of the images represents in false colors the cyan fluorescent protein (CFP) fluorescence lifetime, ranging from 0 to 3 ns. Spherical shapes with a very short fluorescence lifetime reveal chloroplast autofluorescence.

This hypothesis was supported by a FRET-FLIM protein-protein interaction experiment which did not reveal any significant interaction between VvLYK1-2_CFP and VvLYK5-1-YFP before or after chitin treatment (**Figure S9**). FRET-FLIM measurements performed for VvLYK1-1_CFP and VvLYK5-1_YFP did not reveal any interaction between these receptors at the basal state (**Figure 6**). However, 30 min after chitin treatment, a significant decrease of CFP fluorescence lifetime (τ_1_) was observed when VvLYK1-1_CFP was co-transformed with VvLYK5-1_YFP, with a recorded mean τ_1_ decreasing from 2.13 ns for the donor when expressed alone to 1.88 ns when co-expressed with the VvLYK5-1_YFP acceptor (E = 11.70%; p-value< 0.001, **Figure 6**). To note, no interaction between VvLYK1-2_CFP or VvLYK1-1_CFP and VvLYK5-2_YFP before or after chitin treatment could be demonstrated (**Figure S9**, **Figure 6**). Altogether, these results reveal that only VvLYK1-1_CFP and VvLYK5-1_YFP can physically interact upon chitin perception.

## 4 Discussion

Chitin and chitosan are two well-known invasion patterns (IPs) that elicit defense responses in a large range of plant species including *Vitis vinifera* (Felix et al., 1998; Shibuya and Minami, 2001; Kaku et al., 2006; Miya et al., 2007; Sharif et al., 2018; Brulé et al., 2019). In grapevine, both chitin and chitosan oligomers led to the activation of defense mechanisms including the activation of the MAPKs cascade and the expression of defense genes which ultimately confer an increased resistance against grey mold and downy mildew (Brulé et al., 2019). However, the mechanism by which perception of these IPs takes place in grapevine cells is still not well understood. Part of the reason for this may be the complexity of the grapevine genome. Indeed, contrary to *Arabidopsis thaliana* from the *Brassicaceae* family that underwent diploidization and chromosome fusions since its evolutive divergence from the polyploid dicotyledonous ancestor (Blanc et al., 2003; Mandáková et al., 2010), *Vitis vinifera* is still considered as a paleo-hexaploid organism (Jaillon et al., 2007). This is reflected in the number of *LYKs* genes in each species, *i.e.* 5 members in *A. thaliana* (Wan et al., 2008) compared to 16 predicted genes in *V. vinifera*. As AtLYK5 has been described as the main chitin receptor in *A. thaliana*, we searched for closely related genes in the *V. vinifera* genome. Of the two putative grapevine orthologs identified, named *VvLYK5-1* and *VvLYK5-2*, only *VvLYK5-1* shows an increased expression in leaves after inoculation with fungal pathogens and a transient expression 1h after treatment of grapevine cells with chitin oligomer, suggesting a fast production of VvLYK5-1 receptors upon the perception of this fungal IP.

After transformation of the *A. thaliana lyk4/5* double mutant, which is completely deficient in COS-triggered immune responses, with the grapevine *AtLYK5* orthologs, we were able to demonstrate that expression of *VvLYK5-1* was sufficient to restore chitin-triggered immune responses. Indeed, both early (*i.e.* MAPKs activation and defense gene expression) and late (*i.e.* callose deposition) defense reactions were restored after chitin oligomer treatment in the *atlyk4/5-p35S::VvLYK5-1* lines, with levels similar to WT plants. Interestingly, we could only demonstrate fully restored immunity in response to chitin oligomer treatment as none of the MAPK activation nor *FRK1* gene expression was significantly restored after chitosan treatment with the same DP. These results suggest that contrary to the two previously identified grapevine COS receptors VvLYK1-1/-2 which restored chitin and chitosan defense responses, VvLYK5-1 seems to be more specific to GlcNAc residues of chitin than to the deacetylated GlcN present in chitosan oligomers. Moreover, the restoration of penetration resistance to *E. necator* in the *atlyk4/5* double mutant after transformation with the *VvLYK5-1* gene suggests that *VvLYK5-1* contributes to the basal resistance of grapevine cells to this fungal pathogen, probably by recognizing chitin fragments released during penetration attempts.

In the current mechanism of chitin perception in the model plant *A. thaliana*, AtLYK5 is proposed to form homodimers or heterodimers with AtLYK4 and/or AtLYM2 at the basal state (Cheval et al., 2020). After chitin binding to the LysM2 domain of AtLYK5, this receptor forms a complex with AtLYK1 (Cao et al., 2014), concomitantly with the dissociation of AtLYK1 from its proposed negative regulator AtLIK1 (Le et al., 2014). Although no direct interaction could be demonstrated with AtLYK1, AtLYK4 is proposed to stabilize the complex by interacting with AtLYK5 (Xue et al., 2019). The interaction between AtLYK5 and AtLYK1 subsequently induces auto-phosphorylation of AtLYK1 (Liu et al., 2012b), in turn activating AtLYK5 by trans-phosphorylation (Cao et al., 2014; Erwig et al., 2017; Gubaeva et al., 2018). Additional AtLYK5 phosphorylation, presumably driven by the cytosolic protein kinases AtCPK5 and AtCPK6 (Huang et al., 2020), would then lead to downstream signaling events. Finally, after signal transduction and defense gene expression, phosphorylated AtLYK5 (and AtLYK4) would dissociate from AtLYK1 and be internalized into endosomes to regulate chitin signaling (Erwig et al., 2017). As VvLYK5-1 does not appear to possess a functional kinase domain, similarly to AtLYK5, we propose a similar signal transduction pathway for grapevine, initiated at the plasma membrane by chitin-induced interaction of VvLYK5-1 with one of the two previously identified grapevine AtLYK1 orthologs known to mediate chitin-induced immunity (Brulé et al., 2019). Our FRET-FLIM experiments revealed an *in vivo* interaction between VvLYK1-1 and VvLYK5-1 after chitin oligomer treatment, in agreement with the Arabidopsis chitin perception model previously described (Cao et al., 2014). Furthermore, because of its ubiquitous basal expression and its interaction with VvLYK5-1 during the chitin-mediated signal transduction, we propose that VvLYK1-1 might be the grapevine LYK co-receptor that could also integrate perception of other IPs by interacting with corresponding receptors, as it was shown for AtLYK1 and OsCERK1 in Arabidopsis and rice, respectively (Shimizu et al., 2010; Willmann et al., 2011; Liu et al., 2012a; Liu et al., 2013; Yang et al., 2022).

Despite the strong similarity between the protein sequences of VvLYK5-1 and VvLYK5-2, we could not demonstrate any implication of the latter in chitin or chitosan oligomers perception. Intriguingly, when expressed in the *atlyk4/5* double mutant, VvLYK5-2 could restore penetration resistance in epidermal cells to powdery mildew. Considering its strong expression in leaf tissues and these observations, VvLYK5-2 might thus participate in the perception of other IPs. Since immune responses induced by the plant cell wall derived mixed-linked β-1,3/1,4-glucans are altered in *atcerk1* and *atlyk4/5* double mutants (Rebaque et al., 2021), it is tempting to propose a role for VvLYK5-2 in the perception of other oligosaccharidic IPs, that might be released after degradation of the cell walls during fungal penetration in its attempt to colonize plant tissues.

Browsing through the *STRING* database (Szklarczyk et al., 2019) to explore putative VvLYK5-1 and VvLYK5-2 interaction partners (**Figure S7**), we also found a predicted interaction with a kinase-associated protein phosphatase (VvKAPP2C), which is known to interact with other RLKs to negatively regulate defense responses (Rienties et al., 2005; Park et al., 2008; Couto et al., 2016; Gramegna et al., 2016). Moreover, VvLYK5-1 and VvLYK5-2 were predicted to interact with the LysM receptor-like protein VvLYM2-1, which Arabidopsis orthologous (AtLYM2) has been described to mediate CERK1-independent chitin-mediated regulation of the symplastic continuity and resistance to fungal pathogens (Faulkner et al., 2013; Narusaka et al., 2013; Cheval et al., 2020). Although chitin perception is just starting to be studied in *V. vinifera*, it is tempting to propose a perception model similar to that described in rice and Arabidopsis where a co-receptor (OsCERK1 or AtCERK1) interacts with different ligand-binding receptors for inducing chitin or peptidoglycan signaling (Shimizu et al., 2010; Willmann et al., 2011; Cao et al., 2014; Cheval et al., 2020). In this proposed model, VvLYK1-1 and VvLYK5-1 would not interact in the basal state, presumably to allow VvLYK1-1 to interact as co-receptor with other RLK/RLPs, or simply to avoid inappropriate activation of defense reactions triggered by the VvLYK1-1/VvLYK5-1 complex. One or several VvLYK5-1 proteins (according to the length of the chitin fragment) could bind chitin oligomers then interact with the VvLYK1-1 co-receptor to transduce the signal and lead to chitin-induced immunity. Moreover, to allow for regulation of chitin-induced signaling, this supramolecular receptor complex may later be deactivated by protein phosphatases and/or receptor internalization (Erwig et al., 2017). As demonstrated for AtLYM2 in plasmodesmata (Cheval et al., 2020), different complexes are also expected to be formed in particular cellular compartments, and specific tissues or organs.

Deciphering the complete mechanism of chitin and chitosan perception in grapevine would be of great interest to agriculture as it could be used in selection programs to develop grapevine varieties with durable resistance against fungal pathogens and improved the efficacy of commercialized COS-containing biocontrol products. Together with the comprehension of tissue-specific expression patterns, deciphering the role of the different members of the *VvLYK* multigene family would be useful to understand how gene duplications have influenced plant evolution and how closely related IPs are distinguished to induce appropriate defense or signaling pathways.

## 5 Accession numbers

*Vitis vinifera* cv. Marselan sequences: *VvLYK1-1* (OP498352), *VvLYK5-1* (OP498353), *VvLYK5-2* (OP498354) have been deposited to GenBank and will be released after publication.

## Data availability statement

The datasets presented in this study can be found in online repositories. The names of the repository/repositories and accession number(s) can be found below: https://www.ncbi.nlm.nih.gov/genbank/, OP498352 https://www.ncbi.nlm.nih.gov/genbank/, OP498353 https://www.ncbi.nlm.nih.gov/genbank/, OP498354.

## Author contributions

TR performed most of the experiments, analyzed the data and wrote the article. M-CH and BP led the project, supervised and complemented the writing. TM performed and analyzed the qPCR experiments on grapevine cells. DL performed subcellular localization and protein immunoblotting assays. BL selected the first line of transgenic seeds. AJ identify the *atlyk4/5* increased sensitivity to *E. necator* compared to the WT. PW provided technical assistance and helped to interpret data on FRET-FLIM experiments. AG and ID revised the manuscript. All authors contributed to the article and approved the submitted version.
