# Supplementary material for: The grapevine LysM receptor-like kinase VvLYK5-1 recognizes chitin oligomers through its association with VvLYK1-1
Source: Front Plant Sci. 2023 Feb 2;14:1130782. doi: 10.3389/fpls.2023.1130782 (PMC9932513; doi:10.3389/fpls.2023.1130782)
Supplement: Supplementary file 1 [file DataSheet_1.pdf]

Supplementary Figures

A

B C

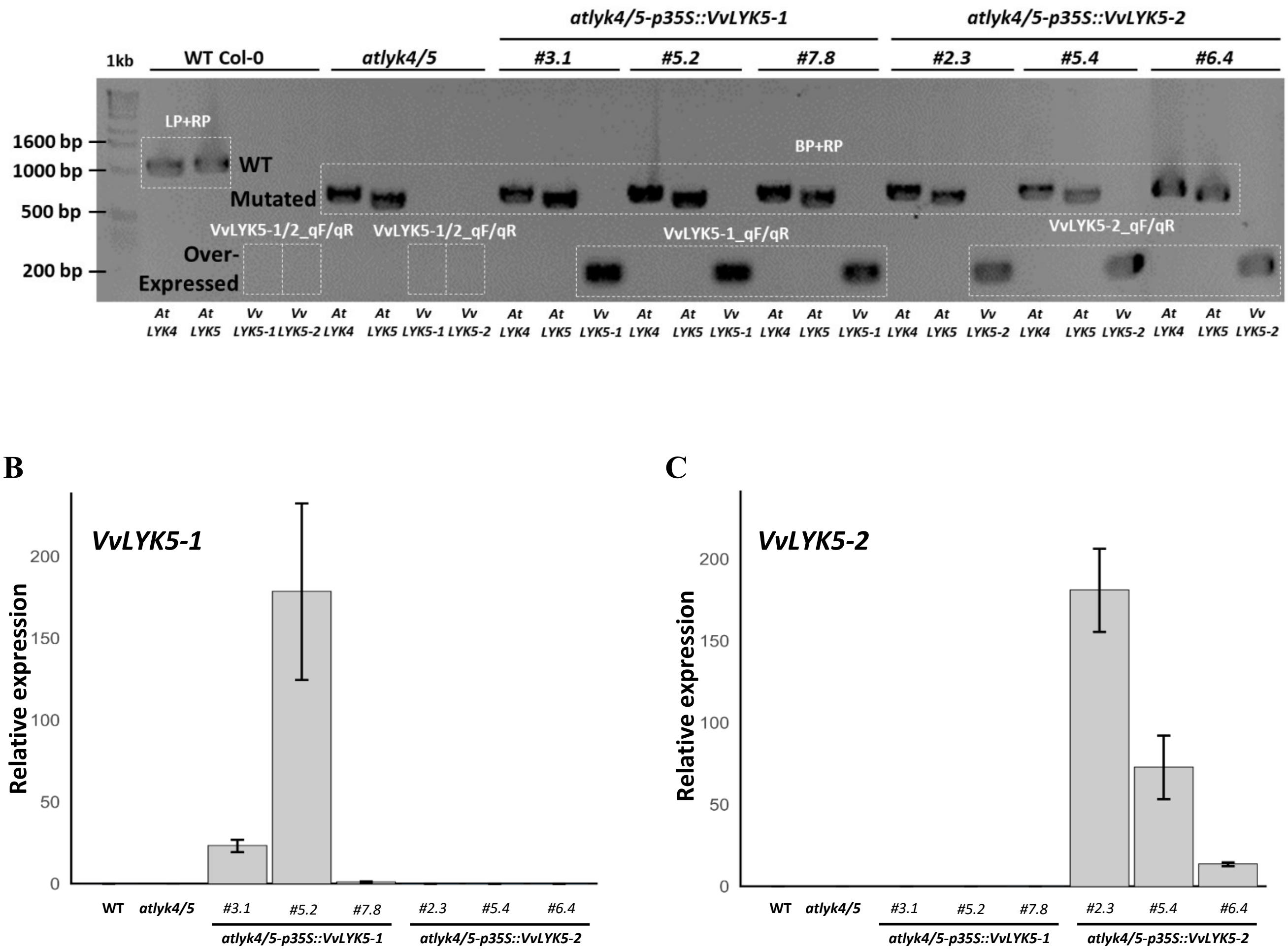

Figure S1. Characterization of the transgenic lines. (**A**) Genotyping of the lines used in the study. Polymerase chain reaction (PCR) was performed on genomic DNA to check the absence or the presence of the T-DNA in the wild-type (WT) and the *atlyk4/5* double mutant or transgenic lines to detect the presence of the grapevine transgenes. Primers used are listed in the supplemental table S1 and have been designed using the *T-DNA Primer Design* tool of the *SIGnAL* website (http://signal.salk.edu/tdnaprimers.2.html). Expected sizes with the LP + RP primers were close to 1100 bp for the WT alleles of *AtLYK4* and *AtLYK5*, respectively. In the Arabidopsis double mutant *atlyk4/*5, PCR products obtained with primers BP + RP were expected at 524-824 bp and 512-812 bp for the T-DNA insertion in the *atlyk4* or *atlyk5* mutated gene, respectively. The presence of transgenes of *VvLYK5-1* and *VvLYK5-2* in the *atlyk4/5* double mutant was checked using the "qPCR" primers used to amplify a region of approximately 200 bp in each transcript. Negative controls were performed on untransformed WT Col-0. (**B, C**) Quantification of the expression of the grapevine transgenes *VvLYK5-1* (**B**) and *VvLYK5-2* (**C**) detected by qPCR. The mean of efficiency-weighted $C_q^{(w)}$ values from technical duplicates of the water control-treated samples were normalized by the $C_q^{(w)}$ average of two housekeeping genes (*AtRHIP1* and *AtPTB1*), set as 1. Data represent the mean relative expression ± SE of four independent experiments.

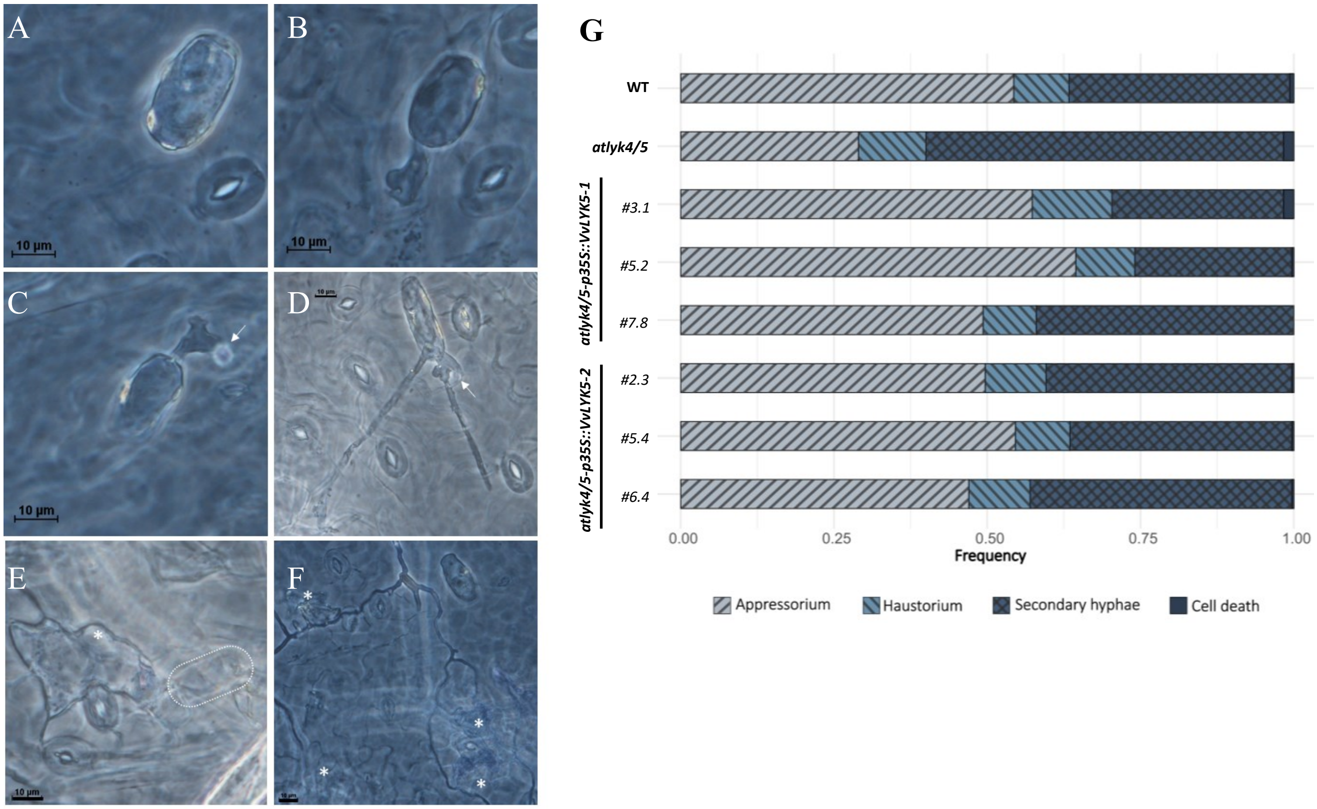

Figure S2. Scoring method for evaluating the resistance to *E. necator*. (**A-F**) Pictures representing different classes : (**A**) Ungerminated spore; (**B**) Spore with appressorium; (**C**) Spore with appressorium and haustorium; (**D**) Spore with secondary hyphae; (**E-F**) Spore inducing cell death. Haustoria have been labeled with arrows, dead cells with asterisks and an out-of-focus spore has been circled with a dashed line. For scoring, only germinated spores were considered. Among them, those showing only an appressorium are evaluated as failing to penetrate the plant cells whereas others (*i.e.* with a haustorium, secondary hyphae, or inducing cell death) are evaluated as having well penetrated inside the cells. Scale bars indicate 10 µm. (**G**) Scoring detail of Fig. 5. Each data point represents the mean of three independent experiments.

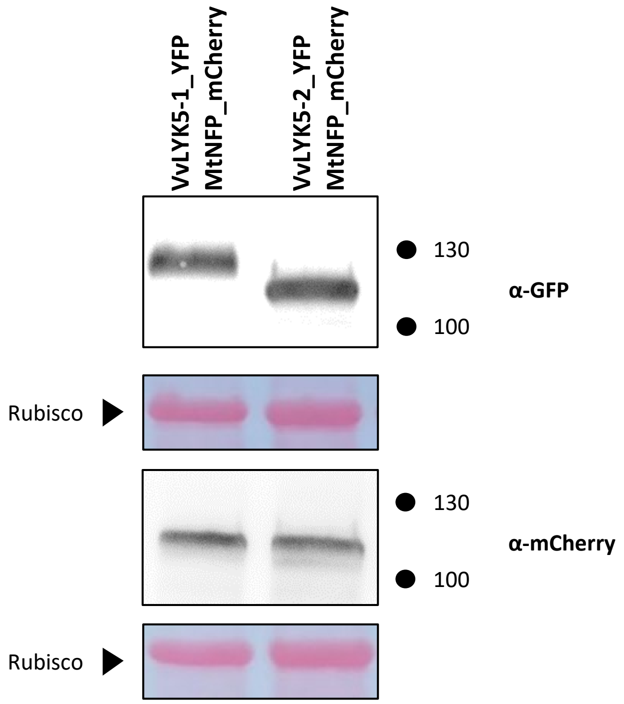

Figure S3. Immunodetection of VvLYK5-1_YFP and VvLYK5-2_YFP during their subcellular localization in *Nicotiana benthamiana* leaves. The integrity of the fusion proteins VvLYK5-1_YFP and VvLYK5-2_YFP detected in Fig. 2B was verified by immunoblotting with an antibody raised against GFP which also recognizes YFP. VvLYK5-1_YFP was detected with a slightly higher molecular weight than expected while the VvLYK5-2_YFP and MtNFP_mCherry proteins were detected at the predicted size. The nitrocellulose membranes were stained with Ponceau red serving as loading control.

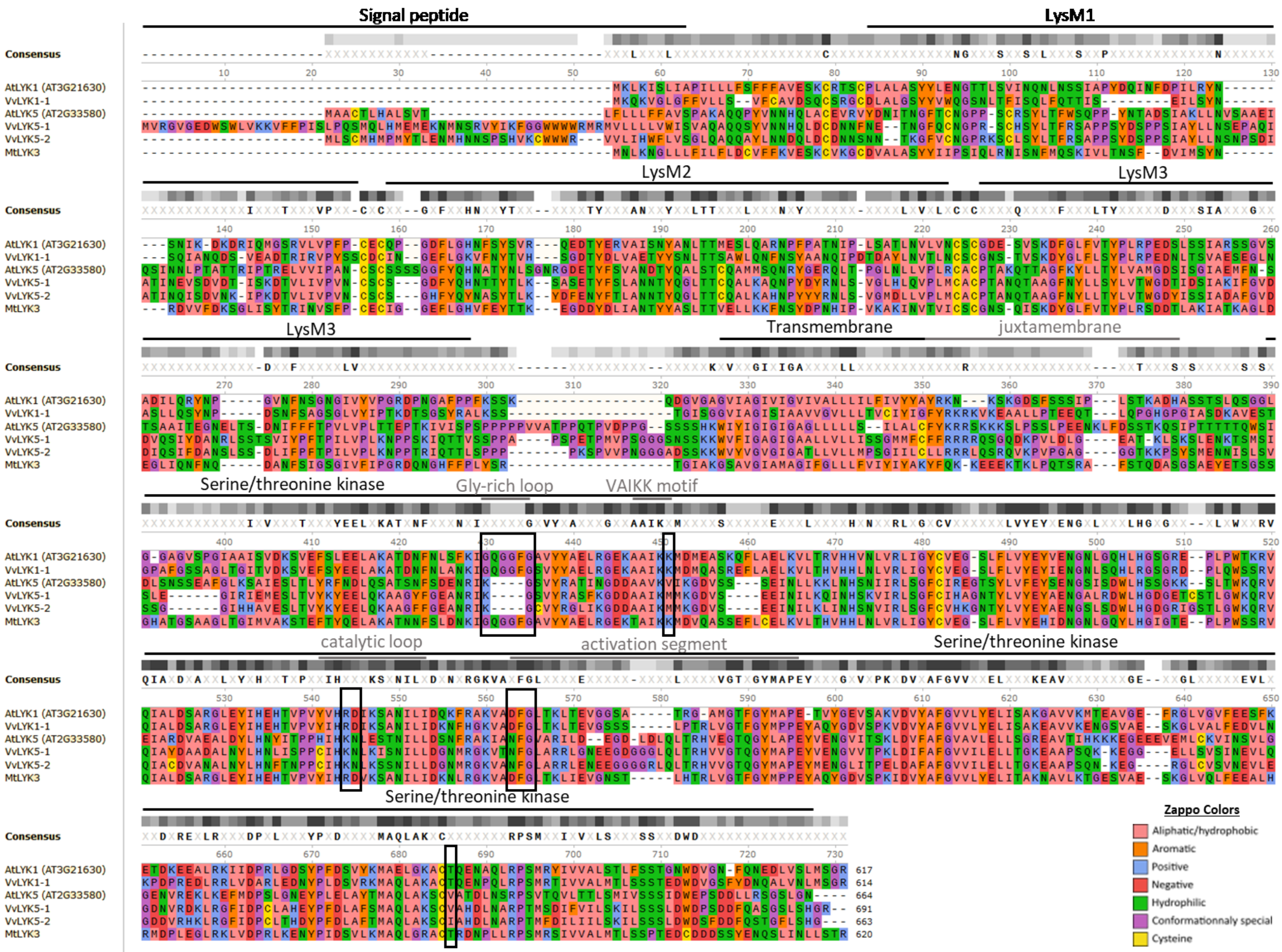

Figure S4. Alignment of the orthologous LYK1 and LYK5 protein sequences from *A. thaliana* and *V. vinifera*. (A) Protein sequences were aligned with *Clustal W*. Sequence conservation is shown as grayscale above the consensus sequence set with a threshold above 50%. Different domain names are indicated on top. Amino acids are highlighted with the Zappo color scheme based on their physico-chemical properties as indicated at the bottom right corner. Well-conserved residues important for the activity of the kinase domain of AtLYK1 in the Glycine-rich loop, the VAIKK motif, the catalytic loop (RD domain), the activation segment (with DFG motif) are indicated by black boxes (Hanks et al., 1988; Johnson et al., 1996; Petutschnig et al., 2010; Klaus-Heisen et al., 2011; Suzuki et al., 2016).

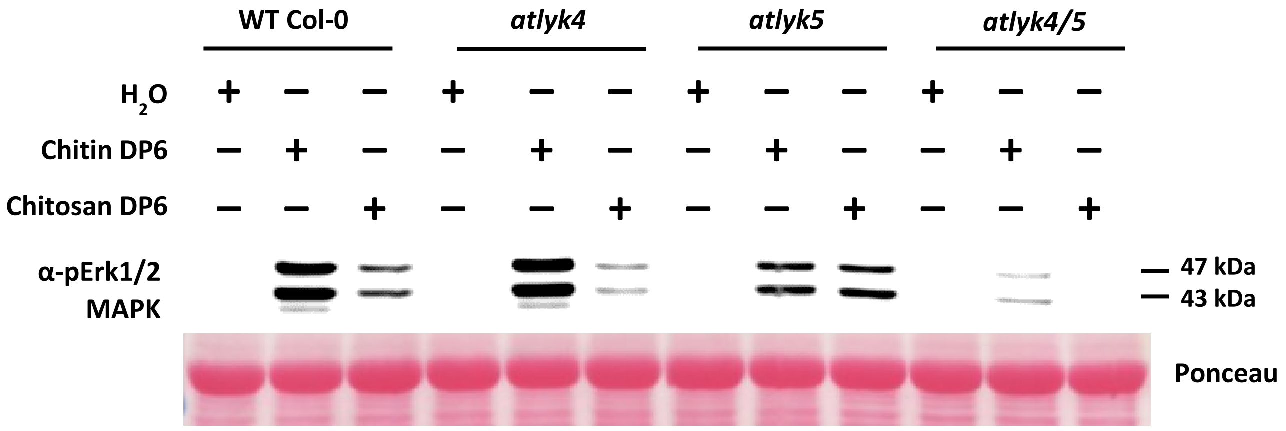

Figure S5. MAPKs activation of *A. thaliana lyk* mutants in response to chitooligosaccharides treatments. The activation of mitogen-activated protein kinases (MAPKs) was detected 10 min after chitin or chitosan treatment (0.1 g/L) by immunoblotting with an antibody raised against the human phosphorylated extracellular regulated protein kinase 1/2 (Erk1/2). Equal loading was confirmed by Ponceau red staining. Similar results were obtained in two independent experiments.

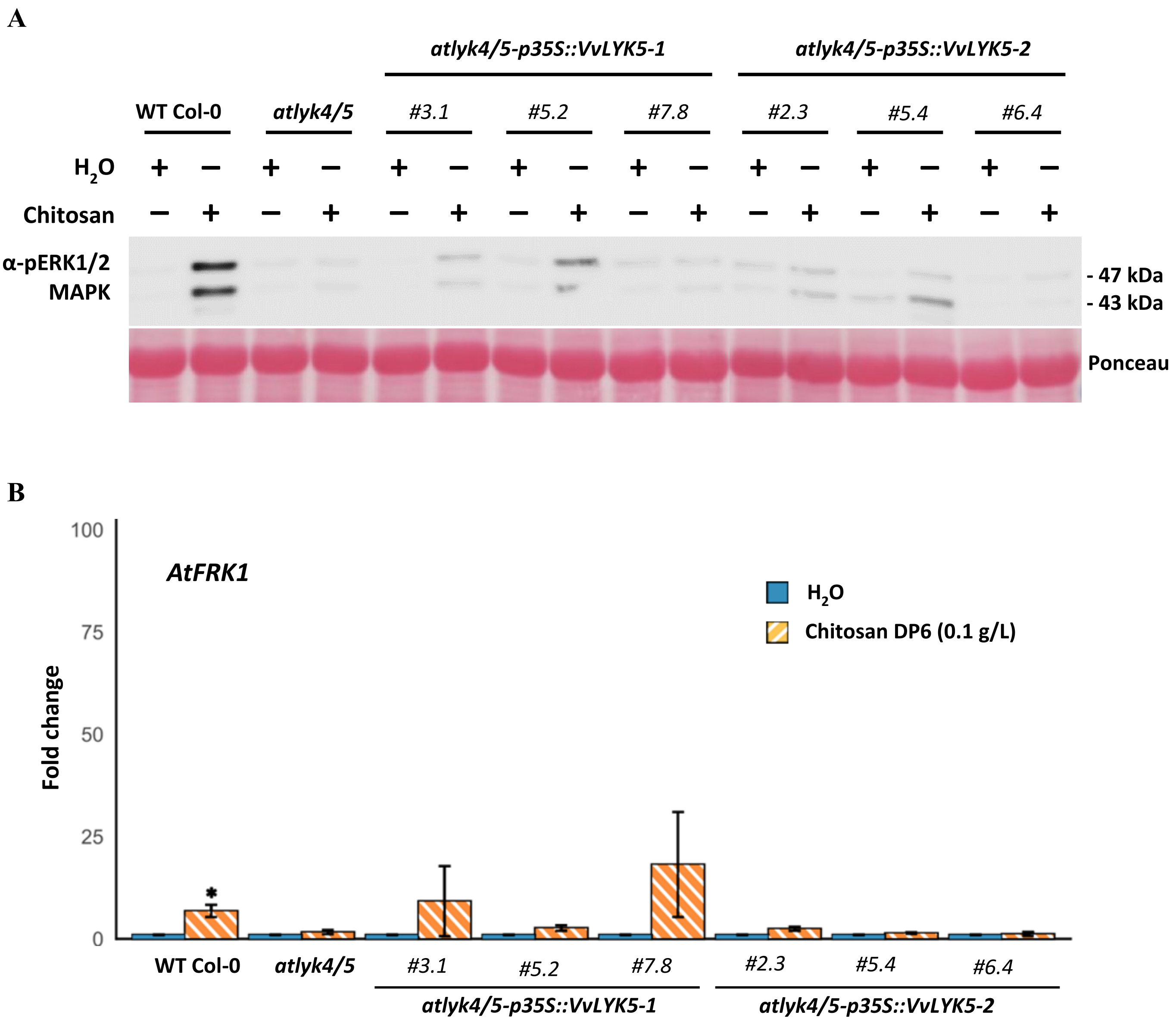

Figure S6. ***VvLYK5-1*** **or** ***VvLYK5-2*** **does not restore early chitosan-induced immune responses in the** ***Arabidopsis thaliana lyk4/5*** **double mutant.** (**A**) The activation of mitogen-activated protein kinases (MAPKs) was detected 10 min after chitosan treatment (0.1 g/L) by immunoblotting with an antibody raised against the human phosphorylated extracellular regulated protein kinase 1/2 (Erk1/2). Homogeneous loading was checked by Ponceau red staining. Similar results were obtained in three independent experiments. (**B**) Fold change in gene expression of a defense gene encoding *flagellin-induced receptor kinase 1 (FRK1; AT2G19190)* measured by qRT-PCR 1h after chitosan (0.1 g/L) or water treatment. Data represent the mean fold-change ± SE from four independent experiments. Means of technical duplicate data (efficiency-weighted $C_q^{(w)}$ values) were normalized by the mean $C_q^{(w)}$ data of two housekeeping genes (*AtRHIP1* and *AtPTB1*) before being normalized to the control treatment. Asterisks indicate a statistically significant difference with the water control (Kruskal-Wallis with Dunn post-hoc test; *, $P < 0.05$ after BH p-value adjustment). WT Col-0, wild-type Columbia-0 ecotype.

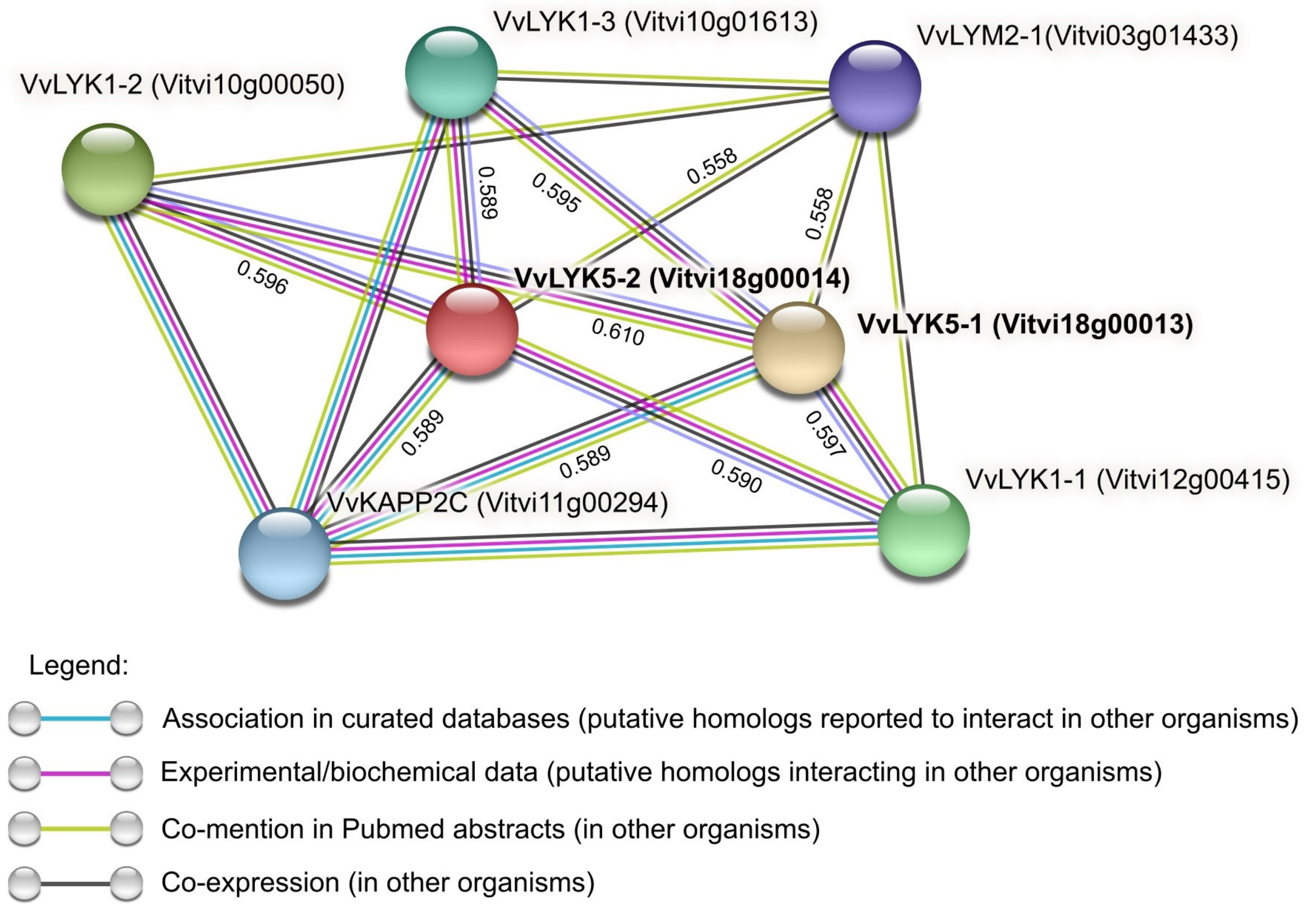

Figure S7. Protein-protein interaction networks of VvLYK5-1 and VvLYK5-2. The analysis was modelled using the *STRING* website (https://string-db.org/). The confidence score ranging from is the approximate probability that a predicted link exists between two proteins according to the *STRING* algorithm (combined score). VvKAPP2C, *V. vinifera* kinase-associated protein phosphatase 2C; VvLYM2-1, *V. vinifera* membrane-anchored LysM receptor-like protein 2-1.

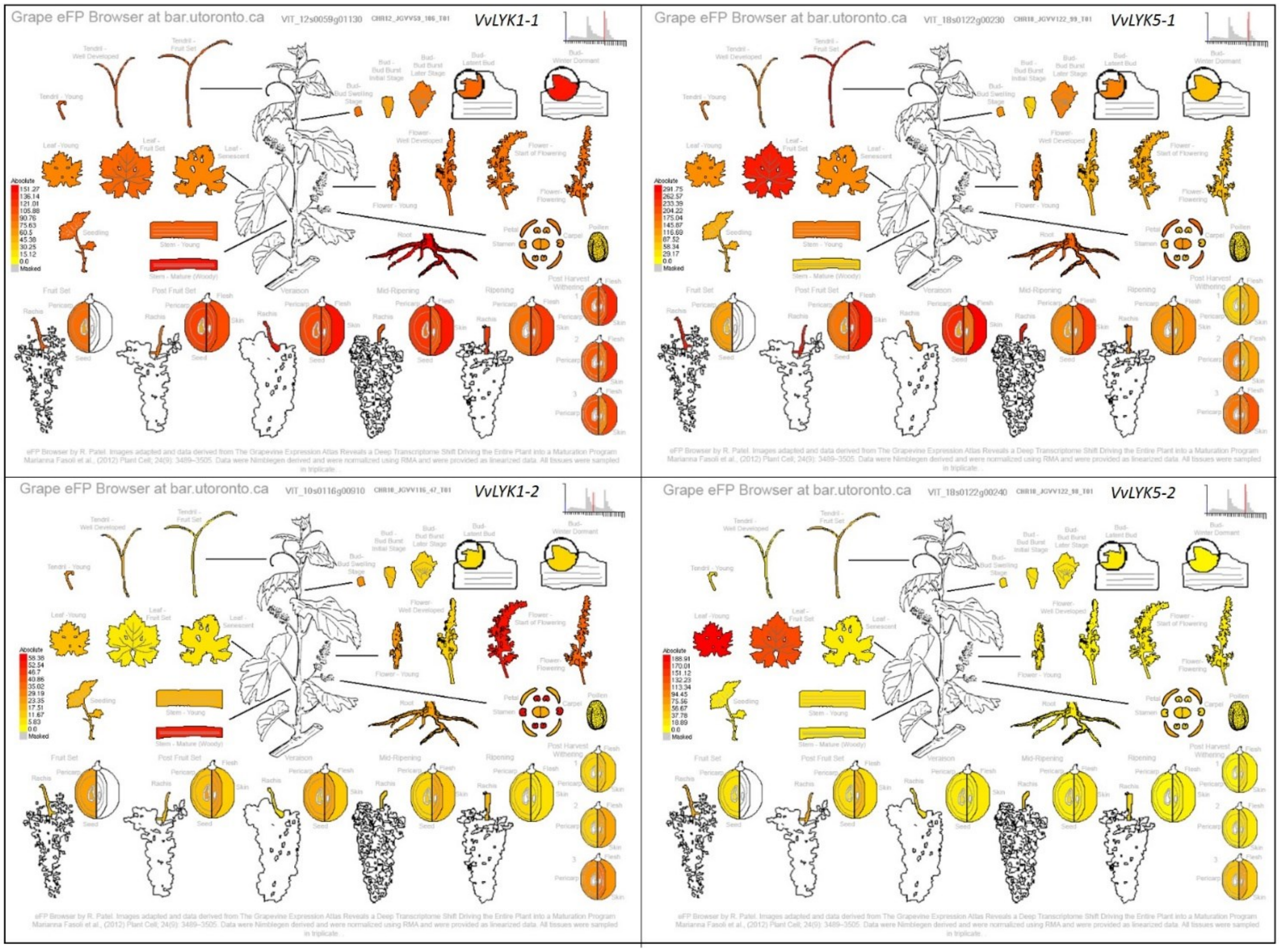

Figure S8. Basal expressions of *VvLYK1-1, VvLYK1-2, VvLYK5-1* and *VvLYK5-2.* Data are absolute expression Nimblegen-derived, normalized using RMA and provided as linearized data extracted. They were obtained from the *Grape eFP Browser* (http://bar.utoronto.ca/efp_grape/cgi-bin/efpWeb.cgi; Fasoli et al., 2012).

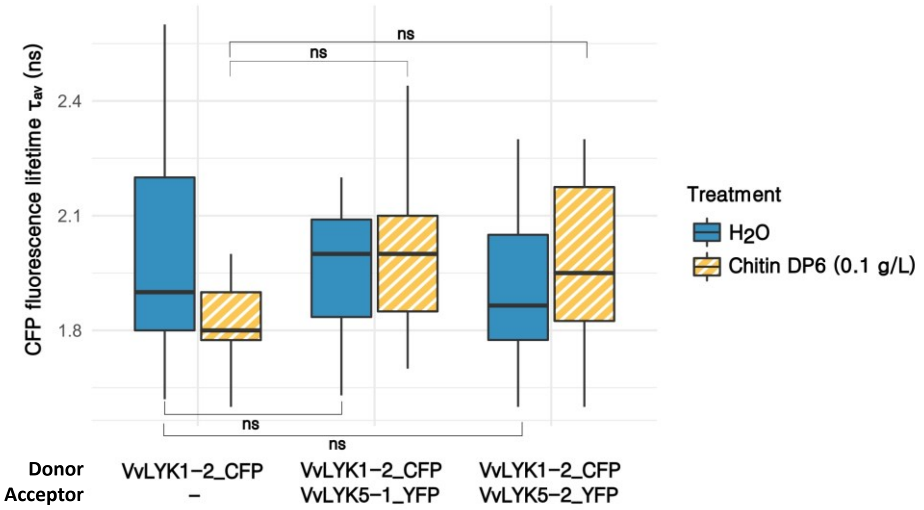

Figure S9. VvLYK1-2 does not seem to interact with VvLYK5-1 and VvLYK5-2. The fluorescence lifetime of the cyan fluorescent protein (CFP) was measured in tobacco leaves (co-)expressing indicated constructs as donor or acceptor, 30 min after infiltration of chitin DP6 (0.1 g/L) or water control. Box plots represent CFP fluorescence-weighted average lifetime (τ_{av}); the box signifies upper and lower quartiles, the line within the box marks the median, and the whiskers represent the maximum and minimum within the 1.5 x interquartile range. The number of ROIs (N) analyzed was > 10. No significant difference emerged from this preliminary experiment (Two-way ANOVA with Tukey post-hoc test).

Supplementary Table S1. Primers used in the study.

Gene	Primer	Sequence (5' → 3')	Usage
VvLYK1-1	VvLYK1-1_full_F	ATGAAACAGAAGGTGGGTTTAGGG	Cloning
VvLYK1-1	VvLYK1-1_full_R	CTACCTTCCAGACATTAGATTGACG	Cloning
VvLYK1-1	VvLYK1-1_fusR	CCTTCCAGACATTAGATTGACGAGG	Cloning
VvLYK1-1	VvLYK1-1_qF	TGGCTTTGTTCGAGGATGTG	qPCR
VvLYK1-1	VvLYK1-1_qR	CGAGTGGGTAGTTATCTTCAAGC	qPCR
VvLYK1-2	VvLYK1-2_full_F	ATGGTCATTTCATCAAACAGCAGG	Cloning
VvLYK1-2	VvLYK1-2_full_R	CTACCTTCCTGACATTAGATTCATC	Cloning
VvLYK1-2	VvLYK1-2_fusR	CCTTCCTGACATTAGATTCATCAGAGC	Cloning
VvLYK1-2	VvLYK1-2_qF	GACCAGAGGCTTGGAGATGA	qPCR
VvLYK1-2	VvLYK1-2_qR	CATCCCAATCTTCGGTTGATGA	qPCR
VvLYK5-1	VvLYK5-1_full_F	ATGGTGAGAGGAGTGGGTGAAGAT	Cloning
VvLYK5-1	VvLYK5-1_full_R	CTATCTACCATGGCTTAGAGAGCCAG	Cloning
VvLYK5-1	VvLYK5-1_fusR	TCTACCATGGCTTAGAGAGCCAGA	Cloning
VvLYK5-1	VvLYK5-1_qF	TAACTGGCAAGGAAGCTGCG	qPCR + Genotyping
VvLYK5-1	VvLYK5-1_qR	GCAACACATGACTTGGCGAG	qPCR + Genotyping
VvLYK5-2	VvLYK5-2_full_F	ATGCTGTCTTGCATGCACATG	Cloning
VvLYK5-2	VvLYK5-2_full_R	TCACCCATGGCTTAGAAAGCC	Cloning
VvLYK5-2	VvLYK5-2_fusR	CCCATGGCTTAGAAAGCCAGT	Cloning
VvLYK5-2	VvLYK5-2_qF	GCTGGCAAGAAGACTGGAGAA	qPCR + Genotyping
VvLYK5-2	VvLYK5-2_qR	CCTTGTTTTGAGAAGGTGCGG	qPCR + Genotyping
VvRPL18B(60S)	VvRPL18B_qF	CCCCTATGCTTTTTGTGGACTTG	qPCR
VvRPL18B(60S)	VvRPL18B_qR	TGCTCGTTTGGGACAATAAACC	qPCR
VvVATP16	VvVATP16_qF	CTTCTCCTGTATGGGAGCTG	qPCR
VvVATP16	VvVATP16_qR	CCATAACAACTGGTACAATCGAC	qPCR
VvVPS54	VvVPS54_qF	GCTGTTTTTGCGGCTTGTGA	qPCR
VvVPS54	VvVPS54_qR	ACCTTCCACCAATCTTCTCCGT	qPCR
-	BP_LB_o8474	ATAATAACGCTGCGGACATCTACATTTT	Genotyping
-	BP_WiscDs_LB	TCCTCGAGTTTCTCCATAATAATGT	Genotyping
AtLYK4	AtLYK4_LP	CATTTTCATCCATCGATGGAC	Genotyping
AtLYK4	AtLYK4_RP	TTCCCTTTCACAACAATCCTG	Genotyping
AtLYK5	AtLYK5_LP	CTTCTTGCCGCTCATACCTC	Genotyping
AtLYK5	AtLYK5_RP	AGCCAATCACTGATCGATCC	Genotyping
AtFRK1	AtFRK1-F	TGAAGGAAGCGGTCAGATTT	qPCR
AtFRK1	AtFRK1-R	CTGACTCATCGTTGGCCTCT	qPCR
AtPTB1	AtPTB1_qF	GATCTGAATGTTAAGGCTTTTAGCG	qPCR
AtPTB1	AtPTB1_qR	GGCTTAGATCAGGAAGTGTATAGTCTCTG	qPCR
AtRHIP1	AtRHIP1_qF	GAGCTGAAGTGGCTTCCATGA	qPCR
AtRHIP1	AtRHIP1_qR	CGTCCGACATACCCATGATCC	qPCR

Supplemental References

Fasoli, M., Dal Santo, S., Zenoni, S., Tornielli, G. B., Farina, L., Zamboni, A., et al. (2012). The grapevine expression atlas reveals a deep transcriptome Shift driving the entire plant into a maturation program. *Plant Cell* 24, 3489–3505. doi: 10.1105/tpc.112.100230.

Hanks, S. K., Quinn, A. M., and Hunter, T. (1988). The protein kinase family: conserved features and deduced phylogeny of the catalytic domains. *Science* 241, 42–52. doi: 10.1126/science.3291115.

Johnson, L. N., Noble, M. E. M., and Owen, D. J. (1996). Active and inactive protein kinases: structural basis for regulation. *Cell* 85, 149–158. doi: 10.1016/S0092-8674(00)81092-2.

Klaus-Heisen, D., Nurisso, A., Pietraszewska-Bogiel, A., Mbengue, M., Camut, S., Timmers, T., et al. (2011). Structure-function similarities between a plant receptor-like kinase and the human interleukin-1 receptor-associated kinase-4. *J Biol Chem* 286, 11202–11210. doi: 10.1074/jbc.M110.186171.

Petutschnig, E. K., Jones, A. M. E., Serazetdinova, L., Lipka, U., and Lipka, V. (2010). The Lysin Motif Receptor-like Kinase (LysM-RLK) CERK1 is a major chitin-binding protein in *Arabidopsis thaliana* and subject to chitin-induced phosphorylation. *Journal of Biological Chemistry* 285, 28902–28911. doi: 10.1074/jbc.M110.116657.

Suzuki, M., Shibuya, M., Shimada, H., Motoyama, N., Nakashima, M., Takahashi, S., et al. (2016). Autophosphorylation of specific threonine and tyrosine residues in Arabidopsis CERK1 is essential for the activation of chitin-induced immune signaling. *Plant and Cell Physiology* 57, 2312–2322. doi: 10.1093/pcp/pcw150.
